# Direct Observation of the Photodegradation of Anthracene and Pyrene Adsorbed onto Mangrove Leaves

**DOI:** 10.1371/journal.pone.0104903

**Published:** 2014-08-21

**Authors:** Ping Wang, Tun-Hua Wu, Yong Zhang

**Affiliations:** 1 School of Environmental Science and Public Health, Wenzhou Medical University, Wenzhou, China; 2 Key Laboratory of Coastal and Wetland Ecosystems (Xiamen University), Ministry of Education, Xiamen, China; 3 School of Information and Engineering, Wenzhou Medical University, Wenzhou, China; 4 State Key Laboratory of Marine Environmental Science (Xiamen University), College of the Environment and Ecology, Xiamen University, Xiamen, China; University of Akron, United States of America

## Abstract

An established synchronous fluorimetry method was used for *in situ* investigation of the photodegradation of pyrene (PYR) and anthracene (ANT) adsorbed onto fresh leaves of the seedlings of two mangrove species, *Aegiceras corniculatum* (L.) Blanco (*Ac*) and *Kandelia obovata* (*Ko*) in multicomponent mixtures (mixture of the ANT and PYR). Experimental results indicated that photodegradation was the main transformation pathway for both ANT and PYR in multicomponent mixtures. The amount of the PAHs volatilizing from the leaf surfaces and entering the inner leaf tissues was negligible. Over a certain period of irradiation time, the photodegradation of both PYR and ANT adsorbed onto the leaves of *Ac* and *Ko* followed first-order kinetics, with faster rates being observed on *Ac* leaves. In addition, the photodegradation rate of PYR on the leaves of the mangrove species in multicomponent mixtures was much slower than that of adsorbed ANT. Compared with the PAHs adsorbed as single component, the photodegradation rate of ANT adsorbed in multicomponent mixtures was slower, while that of PYR was faster. Moreover, the photodegradation of PYR and ANT dissolved in water in multicomponent mixtures was investigated for comparison. The photodegradation rate on leaves was much slower than in water. Therefore, the physical-chemical properties of the substrate may strongly influence the photodegradation rate of adsorbed PAHs.

## Introduction

Over 80% of the earth's terrestrial surface is covered by vegetation [1]. Vegetation plays a key role in the environmental fate of many polycyclic aromatic hydrocarbons (PAHs) [2]. Furthermore, leaf surfaces are covered with a complex lipid cuticle that can accumulate hydrophobic organic pollutants from the atmosphere [3]. PAHs are widely distributed persistent organic pollutants that are generated by natural combustion processes as well as by human activities [4], [5]. These micropollutants in the environment pose a potential threat to aquatic organisms and humans because of their toxic, carcinogenic and mutagenic properties [6]. PAHs are ubiquitous in natural substances such as plants, soil, sediment, water and air [7]–[9]. Thus, it is important to study the transportation and transformation of PAHs in the environment. Many reports demonstrate that most PAHs in the environment biodegrade slowly and with difficulty because of their low water solubility [10]. Additionally, it has been shown that many PAHs exhibit photo-induced toxicity [11] and that the delocalized π bond of PAHs can absorb the visible and ultraviolet components of sunlight [12]. Therefore, photodegradation might represent an important transformation pathway for PAHs in the environment. Investigations in this field have recently intensified and have generated some solid conclusions. At present, most studies on the photodegradation of PAHs have focused on PAHs in a liquid medium or adsorbed onto solid particles [13]–[16]. Only a few studies have addressed the photodegradation of PAHs adsorbed onto vegetation, although the photodegradation of PAHs adsorbed onto plant surfaces (especially leaves) plays an important role in their transfer from the atmosphere to the food chain [17]–[19].

Mangrove wetlands, as a buffer in estuaries, act as both a sink and a source of PAHs in coastal ecosystems [20]–[22]. Mangroves can accumulate lipophilic PAHs from the atmosphere because of the thick waxy or lipidic layers on the surfaces of their leaves [23]. Ke et al. argued that photodegradation is an important method for removing the PAHs adsorbed onto mangrove leaves after an oil spill accident [24]. Wang et al. and Niu et al. found that the photodegradation of some PAHs adsorbed onto spruce or pine needles might play a significant role in their environmental behavior. Therefore, it is necessary to investigate the photochemical behavior of PAHs absorbed onto mangrove leaves. However, previous research on the photodegradation of PAHs adsorbed onto plant leaves has mostly been conducted by entirely destroying the sample. Thus, these traditional methods are not compatible with the direct study of the photodegradation of PAHs adsorbed onto leaves. Moreover, in a long-term study, the quantity of PAHs volatilized from the leaf surfaces and entering the inner leaf tissues may not be negligible [17], [19]. Optical fibers showing high light focalization, low weights and small sizes have been used for the direct investigation of PAHs adsorbed onto solid substrates [25]–[27]. In our group, a fiber-optic fluorimetry has also been established for *in situ* investigation of the photodegradation of fluoranthene adsorbed onto the leaves of three mangrove species [23], [28]. Nevertheless, the mechanisms underlying the photodegradation of PAHs adsorbed onto mangrove leaves have not been fully explored. Our previous research sought to elucidate the environmental behaviors of PAHs adsorbed onto mangrove leaves using an established synchronous fluorimetry combined with a fluorescence spectrophotometer and an optical fiber to directly detect PYR and ANT adsorbed onto mangrove leaves [29]. The photodegradation of PYR and ANT adsorbed onto the leaves of two mangrove species as single component have also been studied in our laboratory [30]. However, the environmental behaviors of PAHs are very complex in the field. For example, many different types of PAHs from different sources might exist on the leaf surfaces. Because of the effects of complex factors mentioned above or others, it is difficult for *in situ* investigation the environmental behaviors of PAHs adsorbed on leaf surfaces in the field. In addition, only a few studies involve the photolytic behaviors of PAHs adsorbed on vegetation. Thus, preliminary experiments could be carried out in lab at first. And simple experimental conditions were set for study of the possible photolysis processes of some PAHs on the typical mangrove leaves. To further develop this line of research, synchronous fluorimetry was used for *in situ* studying the photodegradation of PYR and ANT adsorbed onto the leaves of two mangrove species in multicomponent mixtures. Compared with the photodegration of the PAHs adsorbed on mangrove leaves in single component, the further studies for investigation the photodegration of the PAHs adsorbed on mangrove leaves in multicomponent mixtures are more representative of real conditions for most PAHs in the environment. Seedlings of *Aegiceras corniculatum* (L.) Blanco (*Ac*) and *Kandelia obovata* (*Ko*), two of the most widespread mangrove species in China, were selected for this study, and the photodegradation of equal quantities of PYR and ANT in water as single compounds and in multicomponent mixtures was also investigated for comparison.

## Materials and Methods

### Preparation of PAH solution

Stock solutions of PYR and ANT (Aldrich, purity>99%, USA) were obtained based on the method described in our previous studies [22], [29]. Working solutions of PYR and ANT were prepared by diluting the stock solutions with acetone just prior to being used.

### Sample collection

According to our previous studies and others, the mature viviparous hypocotyls of *Ko* when off the trees could be collected for cultivation, and the non-viviparous seedlings of *Ac* who are least one-year old could be taken away from the forest and cultivated for further experiments [30], [31]. Therefore, mature mangrove hypocotyls of *Ko* and 1-year-old *Ac* seedlings were collected at the Longhai mangrove reserve in Zhangzhou, Fujian, China (longitude: 24°29′3″, latitude: 118°5′59″, altitude: 0 m above sea level). This mangrove forest is wild and does not belong to any individuals or organizations. Thus, no specific permissions were required for these activities. Only a few mangrove seedlings were collected for the experiment, which did not involve endangered or protected species or vertebrates. Hypocotyls of *Ko* and seedlings of *Ac* of approximately the same size and maturity were sampled and quickly transported to the laboratory for cultivation. The hypocotyls of *Ac* and *Ko* were cultivated on sand (collecting from the mangrove forest) in pots, partially submerged in nutrient Hoagland's solution. For a 12-hour photoperiod, propagules were illuminated by 400-W Na solar lighting. The greenhouse temperature was maintained at 25–28°C. Though, the cultivation conditions in lab were different with that in the field, our studies of past and present have attempted to simulate the living environment of the hypocotyls. The cultivation conditions in lab have their own merits. We all know that the growth of the mangrove hypocotyls in the field might be influenced by many factors. The maturity level and integrity of the hypocotyls living in the same forest might be different. Therefore, the similar maturity of hypocotyls and leaves could not be obtained in the field, which might lead to lower accuracy of the results. Thus, the possible mechanisms of environmental behaviors of the PAHs obtained in the field might be different. Taking into consideration of various factors, the cultivation conditions selected in our studies were comparatively reasonable. Ninety days later, the leaves from the two mangrove species with similar length and fresh weight were collected for the further experiment. After the collection of the leaves, experiments were carried out as soon as possible. Pretreatment of the leaves followed the steps established in our previous studies [29], [30], [31]. The picked fresh mangrove leaves were carefully rinsed with distilled water to remove surface silt. After air drying, six circles of 0.5 cm radius were drawn on the upper surfaces of each leaf with a pencil. The size was the same as the light circle formed by the fiber optical probe. With the use of a micropipette, a certain amount of ANT (0.25 nmol spot^−1^) and PYR (0.25 nmol spot^−1^) acetone solutions (5 µL) were applied as homogeneous layers to the circles of upper leaf surfaces respectively. After evaporation of the acetone from the leaf surfaces at room temperature, a series of similar-sized spots were formed, each spot indicating a sample location.

An ultrasonic cleaning device (Model KQ3200, SM, Kun Shan Ultrasonic Instruments Co., Ltd., China) was utilized to extract the lipid wax from the mangrove leaves. The wax content of the leaves was subsequently quantified using a method from described in previous work [28], [31], [32].

### Photodegradation of PAHs absorbed onto the leaves of the two mangrove species

**Figure 1:**
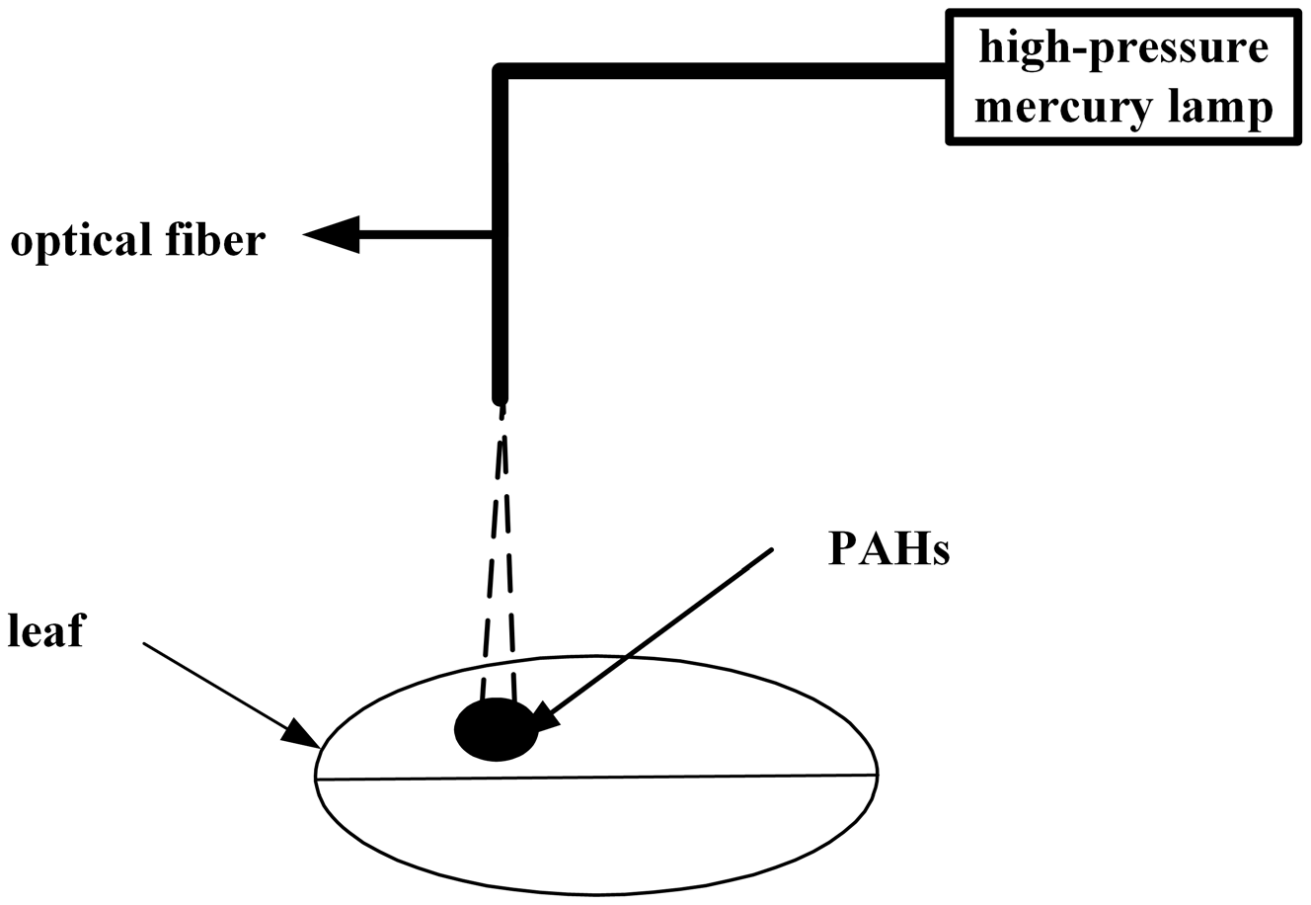
Schematic diagram of the photodegradation of PAHs adsorbed onto the leaves of two mangrove species.

Mangrove leaves with adsorbed PAHs were placed under the optical fiber of a mercury lamp (CHF-XM500 W, Beijing Trusttech, Co., Ltd., China), as shown in Figure 1. Experiments began after the mercury lamp was turned on for approximately 30 minutes. The intensity of illumination, determined with a ZDS-10 illuminometer (Shanghai, China), was maintained at 3.5×10^4^ lx for the entire experiment. The light intensity was controlled by adjusting the height between the mangrove leaf surface and the optical fiber of the mercury lamp. After a certain period of irradiation, the leaves were placed under the fiber-optic probe of the spectrophotometer (Varian, Harbor City, California). In order to get a much clear comparison for the photodegration of ANT and PYR adsorbed on *Ko* and *Ac* leaves as single component and in multicomponent mixtures. According to our previous studies and the pre-experiments, the set irradiation time interval increased gradually (5 nm, 10 nm, 15 nm and 20 nm). Thus, the optimized irradiation of the ANT and PYR adsorbed on the leaves of *Ko* and *Ac* should be set at the same sequence. That is the optimized irradiation time for the PAHs adsorbed onto the *Kc* leaves was set at 0 min, 1 min, 3 min, 7 min, 13 min, 20 min, 30 min, 45 min, 60 min, 80 min, and 100 min, 120 min and 140 min, respectively, and the optimized irradiation time for the adsorbed PAHs on the *Ac* leaves was set at 0 min, 1 min, 3 min, 7 min, 13 min, 20 min, 30 min, 45 min, 60 min, 80 min, 100 min, 120 min and 140 min, respectively [30]. Fluorescence spectra were obtained using the following instrumental setting: excitation and emission slits of 20 and 10 nm, respectively; scan speed of 120 nm min^−1^; PMT voltage of 600 V; and the constant wavelength difference (Δ*λ*) was set at 38 nm. Finally, the fluorescence intensities of the PAHs adsorbed onto the leaves were directly determined. Replicate experiments were conducted for 3 times. To avoid interference from the scattered light, the leaf had to be kept flat, and the angle between the fiber-optic probe of the spectrophotometer and the tested leaf was 45° throughout the experiment.

### Photodegradation of PAHs dissolved in water

To compare and analyze the photodegradation behavior of each PAH in different media, the photodegradation of the same initial amount of PYR and ANT dissolved in water in single-component and multicomponent mixtures was also studied. A photodegradation reactor constructed in our laboratory was used to study the photodegradation of each PAH in water [33]. Using a micropipette, 0.25 nmol of PYR and 0.25 nmol of ANT dissolved in water were added to the reactor, which was then immediately placed under the optical fiber of the mercury lamp. According to our previous studies and the pre-experiments [30], the optimized irradiation time for the ANT in water as single component (0 min, 1 min, 3 min, 6 min, 11 min, 16 min, 21 min, 31 min, 36 min, 41 min, and 56 min) and in multicomponent mixtures (0 min, 1 min, 3 min, 6 min, 11 min, 16 min, 21 min, 31 min, 36 min, 41 min, and 56 min, 66 min, and 76 min) was set, respectively. In addition, the optimized irradiation time for the PYR in water as single component (0 min, 1 min, 3 min, 6 min, 11 min, 16 min, 21 min, 31 min, 36 min, 41 min, 56 min, 66 min, 86 min, 100 min, 120 min) and in multicomponent mixtures (0 min, 1 min, 3 min, 6 min, 11 min, 16 min, 21 min, 31 min, 36 min, 41 min, 56 min, 66 min, 76 min, 86 min, and 96 min) was also set, respectively. To sum up, the set irradiation time of the PAHs in water was almost fixed (mainly 5 nm), which was different with that adsorbed on mangrove leaves (5 nm, 10 nm, 15 nm and 20 nm). Thus, after a defined period of illumination mentioned above, the reactor was placed in the fluorescence spectrophotometer. The fluorescence intensities of the PAHs dissolved in water were ultimately obtained. Because of the small volume of the self-made reactor, the working solutions of PYR and ANT did not require stirring, and we could directly detect the PAHs during photodegradation. Replicate experiments were conducted for 6 times.

### Statistical analysis

In this study, the sample preparations were the same with the method of our previous studies [29]–[31]. And we used four mangrove leaves from each species, yielding a total of 24 locations and 72 measurements for each species. The mean values presented represent the fluorescence intensity of the PAHs adsorbed onto different locations (24 locations from 4 leaves) in the leaves of each mangrove species. Statistical analysis of the variation in fluorescence intensity was performed using the Statistical Package for the Social Science (SPSS), 13.0, for Windows. Significant differences in the results about the mean values of fluorescence intensity obtained by the adsorbed PAHs at different irradiation time were determined using the independent sample t-test. In addition, the confidence interval was utilized for estimate the reliability of the fluorescence intensity of the PAHs adsorbed onto different locations in the leaves of each mangrove species. And the confidence interval was stated at the 95% confidence level (p>0.05 means that no significant difference existed).

When the concentration of a PAH is proportional to its fluorescence intensity, *C_t_* (the PAH concentration at time *t*) and *C_0_* (the initial PAH concentration) can be replaced with *F_t_* (the PAH fluorescence intensity at time *t*) and *F_0_* (the initial PAH fluorescence intensity), respectively. The data were processed based on the same equations as our previous studies [30].

## Results and Discussion

### Fluorescence spectra of PYR and ANT adsorbed onto mangrove leaves

**Figure 2:**
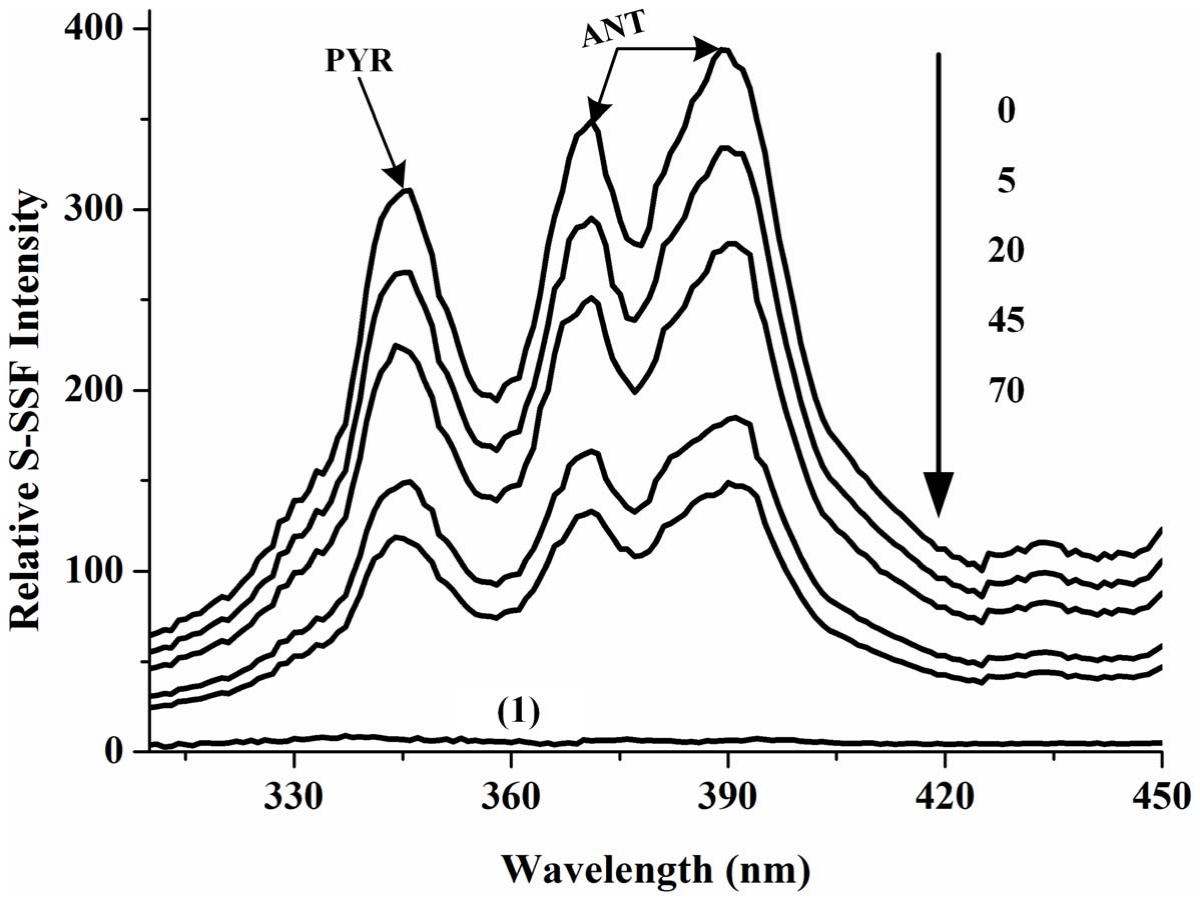
Synchronous fluorescence spectra of PYR and ANT adsorbed onto the leaves of *Ko* in multicomponent mixtures (Δλ = 38 nm), measured at different times during irradiation. The order of the changes in fluorescence intensity over time (at 0 min, 5 min, 20 min, 45 min and 70 min) is indicated by the arrow. Wavelengths of 390 nm and 346 nm were selected for ANT and PYR, respectively. (1): Synchronous fluorescence spectra of the uncontaminated *Ko* leaves.

In previous studies, we established the synchronous fluorimetry method for the direct determination of PYR and ANT adsorbed onto *Ac* and *Ko* leaves both as single compounds and in multicomponent mixtures. Experimental results have indicated that when the optimized scanning wavelength difference (Δλ) was 38 nm, the ANT and PYR adsorbed onto the leaves of two mangrove species as single compounds and in multicomponent mixtures have their own characteristic synchronous fluorescence spectra. The fluorescence spectra of the adsorbed PAHs on mangrove leaves as single component have fluorescence maximums at 369 nm and 387 nm for ANT, and 343 nm for PYR. Thus, the wavelengths of 387 nm and 343 nm were selected for the quantification of ANT and PYR as single component, respectively. In addition, the fluorescence spectra of the PAHs adsorbed on mangrove leaves in multicomponent mixtures showed peaks at 371 nm and 390 nm for ANT, and 346 nm for PYR. Thus, the wavelengths of 390 nm and 346 nm were selected for the quantification of ANT and PYR in multicomponent mixtures, respectively [31]. More recently, the photodegradation of PYR and ANT adsorbed onto the leaves of two mangrove species as a single component has been investigated by our group [30]. However, it is reported that the photodegradation of PAHs are very complex in natural environment. For example, interaction between different types of PAHs might influence the photodegradation of the PAH in multicomponent mixtures. Thus, compared with the PAHs as single component, studies about of the photodegradation of the PAHs in multicomponent mixtures are more representative of real conditions for most PAHs in natural environment. To better understand the photodegradation of these PAHs in multicomponent mixtures, evaluation of the synchronous fluorimetry spectra of the two PAHs and their photodegradation during irradiation is useful. Figure 2 shows the fluorescence spectra of PYR and ANT adsorbed onto the leaves of *Ko* in multicomponent mixtures, measured at different times during irradiation. The relative fluorescence intensities decreased with time. As can be observed in Figure 2, the autofluorescence of *Ko* leaves without PAH was weak and was insufficient to affect the determination of adsorbed PAH in further analyses. In addition, there was no overall change in the shapes of the spectra of the PAHs or the width of the half-wide spectral band. Thus, the photolytic products did not interfere with the detection of adsorbed PYR and ANT during photodegradation (Figure 2). A similar trend was observed during the photodegradation of PYR and ANT adsorbed onto the leaves of *Ac*. Thus, it was concluded that synchronous fluorimetry could be used for the direct study of the photodegradation of PYR and ANT adsorbed onto mangrove leaves in multicomponent mixtures.

### Results of the photodegradation experiments

**Figure 3:**
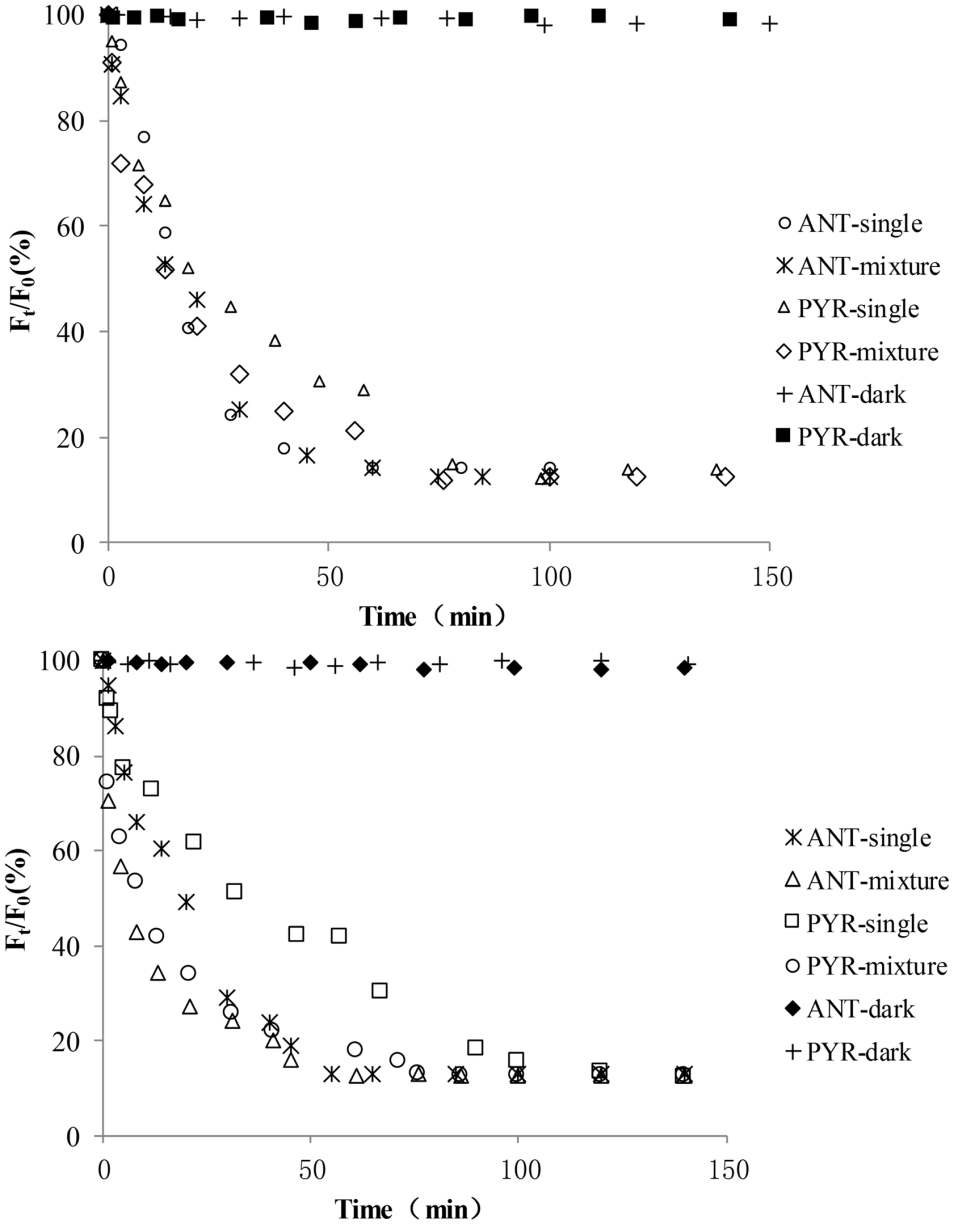
Change in *F_t_/F_0_* with time during the photodegradation of ANT (0.25 nmol spot^−1^) and PYR (0.25 nmol spot^−1^) adsorbed onto *Ko* and *Ac* leaves as single component and in multicomponent mixtures.

**Figure 4:**
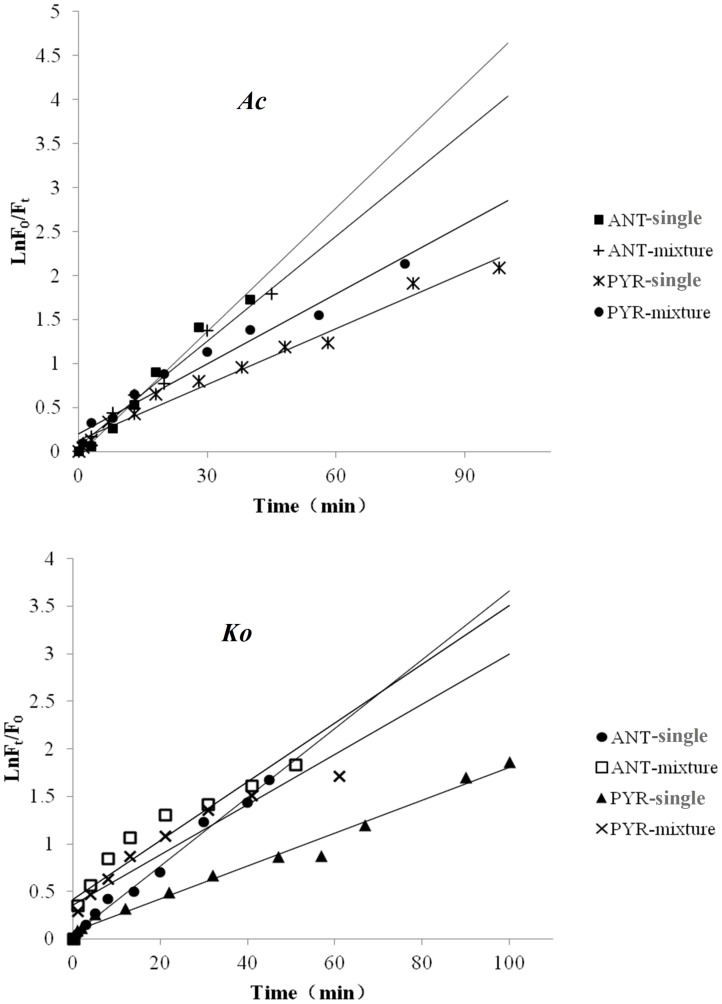
First-order kinetic plots of the photodegradation of ANT (0.25 nmol spot^−1^) and PYR (0.25 nmol spot^−1^) adsorbed onto *Ko* and *Ac* leaves as single component and in multicomponent mixtures.

Volatilization and absorption losses could be the most important pathways for adsorbed PAHs on plant leaves [22]. Therefore, control experiments were carried out in which the leaves were maintained in darkness. The variations in *F_t_*/*F_0_* for PYR and ANT adsorbed onto the leaves of the two mangrove species in multicomponent mixtures were determined over a certain time interval during the photodegradation experiments (Figure 3). Without illumination, *F_t_*/*F_0_* decreased very little as time went on (Figure 3). Hence, photodegradation was found to be the main transformation pathway for PYR and ANT adsorbed onto the mangrove leaves, and the amount of PAH volatilizing from the leaf surfaces and entering the inner leaf tissues could be ignored in the short term. In addition, the relative fluorescence intensities of PYR and ANT as single component are also shown in Figure 3 for comparison [30]. As shown in Figure 3, the curves indicated that the photodegradation of each PAH occurred via multi-decays. Over a given period of time, the photodegradation of both PYR and ANT adsorbed onto the leaves of two mangrove species in multicomponent mixtures basically followed first-order reaction kinetics (Figure 4). In addition, the photodegradation of both PYR and ANT adsorbed onto the leaves of two mangrove species as single component basically followed first-order reaction kinetics in a given period of time (reference 30). Specifically, the given period of time for adsorbed ANT both on the leaves of *Ko* and *Ac* in multicomponent mixtures was from 0 min to 45 min. The given period of time for adsorbed PYR both on the leaves of *Ac* in multicomponent mixtures was from 0 min to 80 min the given period of time for adsorbed PYR on the leaves of *Ko* in multicomponent mixtures was from 0 min to 60 min (Figure 4). The photodegradation rate of the PAHs adsorbed onto *Ac* leaves was faster than that adsorbed onto *Ko* leaves. Moreover, for a given amount of the two substances (0.25 nmol spot^−1^), the photodegradation rate of adsorbed PYR was much lower than that of ANT. Thus, it is a reasonable assumption that the molecular structure of PAHs might be one of the important factors determining their photochemical behavior [34], [35]. The experimental results also showed that for the same initial amount of each PAH, the photodegradation rate was in the order *Ac*>*Ko* (Table 1). It is assumed that the waxes and lipids on plant leaves can absorb UV photons, which might create light-filtering effects [17], [19]. Thus, the number of UV photons striking each PAH adsorbed onto the leaves decreased and thereby reduced the photodegradation rate of each PAH. In this experiment, the leaf-wax content of *Ac* (4.98 mg.g^−1^) was found to be much lower than that of *Ko* (7.63 mg.g^−1^). Thus, the light filtering effect of *Ko* leaves might be stronger than that of *Ac* leaves. Consequently, for the same initial amount of each substance, the number of the UV photons striking each PAH adsorbed onto *Ac* leaves would be higher. Moreover, because of the lower leaf-wax content of *Ac*, the interactions between the adsorbed PAH and *Ac* might become much weaker, making it much easier for the UV photons to strike the adsorbed PAH. These phenomena may act together to explain why the photodegradation rate of the PAHs adsorbed onto *Ac* leaves was faster compared with *Ko* leaves. (Table 1, Table 2). In addition, for both PYR and ANT, the variations in *F_t_*/*F_0_* remained relatively stable when the amount of the adsorbed PAHs on the leaves decreased to approximately 12% of the initial amount (Figure 3). This finding indicates that the photodegradation of both PYR and ANT adsorbed onto the mangrove leaves could only result in partial degradation. In other words, photodegradation of the residual PAH adsorbed onto the mangrove leaves could no longer occur as the illumination time went on. Similar results were obtained regarding the photodegradation of PYR and ANT as single component in our previous work [30]. Identical results were found in studies by Xu *et al.* and Schuler *et al.*, though these authors did not suggest any mechanisms for these findings [36], [37]. Given previous findings by ourselves and others, a small amount of PAH might enter into the inner leaf tissues. These PAHs in the internal leaf tissues cannot absorb UV photons due to the light filtering effects of the leaves [17], [19], [32]. Further research will be required to better understand the possible reasons for these observations.

**Table 1:** Kinetic results for the photodegradation of ANT adsorbed onto the leaves of two typical mangrove species (n = 9) and dissolved in water (n = 9) as single compounds and in multicomponent mixtures.

ANT	Substrate	Calibration carve	Correlation coefficient	*K*	*t* ^1/2^ (min)
single^a^	*Ko*	y^b^ = 3.62×10^−2^x^c^+0.043	0.9952	3.62×10^−2^	19.2
	*Ac*	y = 4.68×10^−2^x−0.036	0.9908	4.68×10^−2^	14.8
	water	y = 5.22×10^−2^x+0.042	0.9960	5.22×10^−2^	13.3
mixture	*Ko*	y = 3.09×10^−2^x+0.417	0.9356	3.09×10^−2^	22.4
	*Ac*	y = 3.98×10^−2^x+0.063	0.9936	3.98×10^−2^	17.4
	water	y = 4.81×10^−2^x−0.017	0.9939	4.81×10^−2^	14.4

a data from our previous studies [30].

b the value of ln(*F*_0_/*F*_t_).

c the illumination time of ANT adsorbed onto mangrove leaves.

**Table 2:** Kinetic results of the photodegradation of PYR adsorbed onto the leaves of two typical mangrove species (n = 9) and dissolved in water (n = 9) as single compounds and in multicomponent mixtures.

PYR	Substrate	Calibration carve	Correlation coefficient	*K*	*t* ^1/2^ min
single^a^	*Ko*	y^b^ = 1.58×10^−2^x^c^+0.114	0.9899	1.58×10^−2^	43.9
	*Ac*	y = 2.13×10^−2^x+0.125	0.9865	2.13×10^−2^	32.5
	water	y = 2.88×10^−2^x+0.088	0.9937	2.88×10^−2^	24.1
mixture	*Ko*	y = 2.61×10^−2^x+0.357	0.9393	2.61×10^−2^	26.6
	*Ac*	y = 2.95×10^−2^x+0.203	0.9815	2.95×10^−2^	23.5
	water	y = 3.46×10^−2^x−0.045	0.9878	3.46×10^−2^	20.0

a data from our previous studies [30].

b the value of ln(*F*_0_/*F*_t_).

c illumination time of the PYR adsorbed onto mangrove leaves.

The experimental results also revealed that compared with PYR and ANT adsorbed onto the leaves of the same mangrove species as single component, the photodegradation rate of ANT in multicomponent mixtures was slower, while that of PYR was faster (Table 1, Table 2). Similar results were found concerning the photodegradation of PYR and ANT in water in multicomponent mixtures (Table 1, Table 2). There are several possible reasons for these results. First, for the same light intensity and irradiation time, the total number of UV photons striking each PAH adsorbed onto the leaves as single component and multicomponent mixtures is the same. Therefore, compared with each PAH as a single compound, the number of UV photons received by each PAH in multicomponent mixtures is lower, which could slow the photodegradation rate of each PAH. Second, anthraquinone is one of the intermediate products of the photodegradation of ANT, and it has also been found to be a photosensitizer for both PYR and ANT [38]. In this experiment, the photodegradation rate of adsorbed ANT was faster compared with adsorbed PYR in multicomponent mixtures (Table 1, Table 2). Hence, in multicomponent mixtures, ANT degraded first and generated some anthraquinone. This anthraquinone might accelerate the photodegradation rate of the adsorbed PYR, and the amount of anthraquinone, as a photosensitizer of adsorbed ANT, would therefore decrease compared with that in the single-component mixture. Thus, the photodegradation rate of the adsorbed ANT would slow down in multicomponent mixtures. However, further studies are needed to confirm this hypothesis.

**Figure 5:**
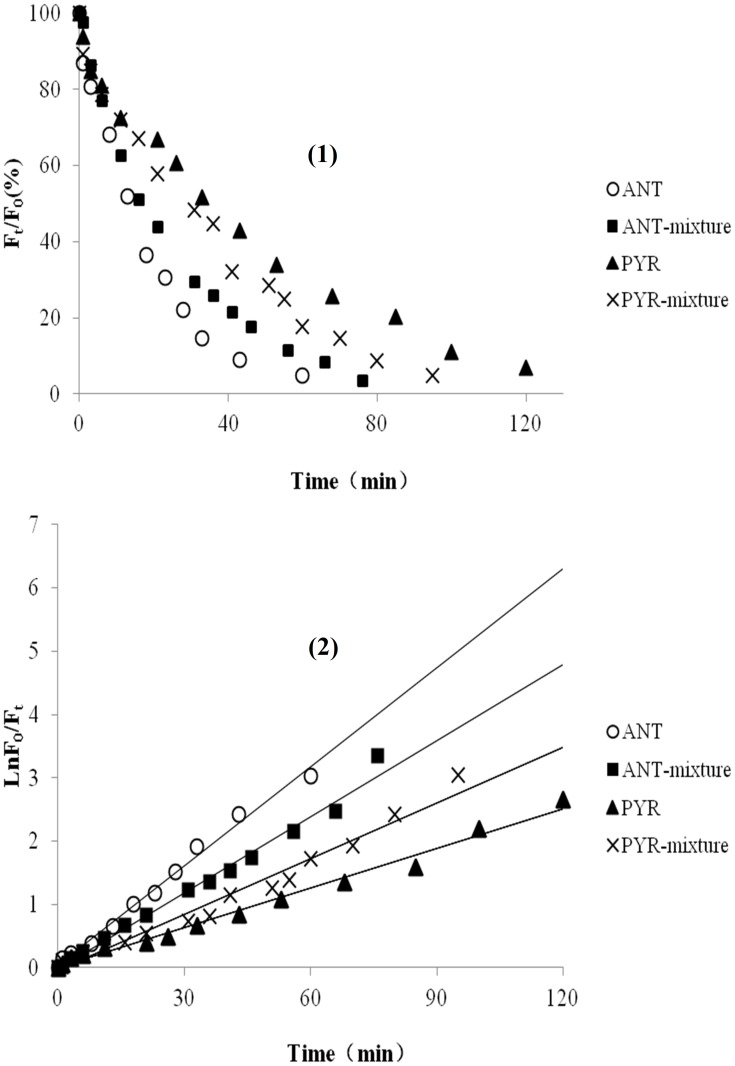
Photodegradations of ANT (0.25 nmol) and PYR (0.25 nmol) in water as single component and in multicomponent mixtures. (1): Change in *F_t_/F_0_* over time during the photodegradation of the two PAHs adsorbed onto the leaves of the two mangrove species. (2): First-order kinetic plots of the photodegradation of the two PAHs adsorbed onto the leaves of the two mangrove species.

Studies show that the polarity of a solvent can significantly affect the photodegradation rate of PAHs in or on that solvent [14]. To further study the possible mechanisms underlying the photodegradation of PAHs adsorbed onto mangrove leaves in multicomponent mixtures, the photodegradation of PYR and ANT dissolved in water in multicomponent mixtures was also investigated for comparison (Figure 5). In this experiment, the synchronous fluorimetry method established in our previous studies was used to determine PYR and ANT levels in water as single component and in multicomponent mixtures [33], [39]. Experimental results showed that the photodegradation of PYR and ANT dissolved in water as single component and multicomponent mixtures followed first-order reaction kinetics (Figure 5). The photodegradation rate of each PAH in water as single component and in multicomponent mixtures was faster compared with the PAHs adsorbed onto mangrove leaves (Table 1, Table 2). There are two possible reasons for this phenomenon. First, the wax layer on the mangrove leaves mainly consists of saturated fatty acids and unsaturated fatty acids, which could reflect or adsorb UV light. Therefore, the number of UV photons striking the adsorbed PAH might decrease and thereby reduce the photodegradation rate [17], [18], [37]. Second, it has been shown that the more polar the solvent, the faster photodegradation is [40], [41]. In the present study, we assessed the PAHs in water, which is polar, and on the mangrove leaf surface, which is nonpolar. Thus, the photodegradation of the PAHs adsorbed onto the leaves was slower than when they were dissolved in water. In addition, it was clearly observed that the relative fluorescence intensities of PYR and ANT in water in multicomponent mixtures decreased almost to zero as the illumination time went on (Figure 5). In other words, PYR and ANT in homogeneous water could be thoroughly degraded within a certain period of time. This result differed from the photodegradation of PYR and ANT adsorbed onto the heterogeneous mangrove leaves. We can speculate that the photodegradation mechanisms for PAHs adsorbed onto mangrove leaves and dissolved in water may differ. Therefore, the photochemical behaviors of PAHs were dependent not only on their molecular structure but also on the physical-chemical properties of the substrate. Further experimental verification is needed.

## Conclusions

In this work, synchronous fluorimetry was shown to be a simple, rapid and easy method for direct investigation of the photodegradation of PYR and ANT adsorbed onto the leaves of *Ac* and *Ko* in multicomponent mixtures. This method may provide a new way to study the mechanisms underlying the phytoremediation of PAHs in mangrove wetlands or in other contaminated media. The photodegradation of both PYR and ANT on the leaves of the studied species followed first-order reaction kinetics. However, the photodegradation processes were different for the same PAHs adsorbed onto the leaves of different mangrove species. In addition, for the same amount of substance, the photodegradation rate of PYR was much slower than that of ANT. Compared with the photodegradation rates in single-component mixtures, the rates in multicomponent mixtures were different. The photodegradation mechanisms of the PAHs adsorbed onto the mangrove leaves, whether in single or multicomponent mixtures, might also be different than the mechanisms for the PAHs is dissolved in water. However, further research is needed to address this conjecture. In this experiment, only the PAHs adsorbed onto the leaf surfaces could be studied, and the PAHs entering into the internal leaf tissues could not be quantified or investigated for further photodegradation. Thus, there are still detailed and comprehensive studies to be carried out in the future. For example, in our group, two-photon laser confocal scanning microscopy has been used for direct visualization of PAHs on and within mangrove leaves. Combining these two methods with some traditional approaches may be helpful for better understanding the environmental behaviors of PAHs in real environments. Therefore, the synchronous fluorimetry method might be valuable in theoretical and practical applications.
